# Clemizole and La^3+^ salts ameliorate angiotensin II‐induced glomerular hyperpermeability in vivo

**DOI:** 10.14814/phy2.14781

**Published:** 2021-05-27

**Authors:** Julia Dolinina, Anna Rippe, Carl M. Öberg

**Affiliations:** ^1^ Department of Nephrology Clinical Sciences Lund Lund University Lund Sweden

**Keywords:** Glomerular permeability, TRPC5, TRPC6

## Abstract

Angiotensin II (Ang II) induces marked, dynamic increases in the permeability of the glomerular filtration barrier (GFB) in rats. After binding to its receptor, Ang II elicits Ca^2+^ influx into cells, mediated by TRPC5 and TRPC6 (transient receptor potential canonical type 5 and 6). Clemizole and La^3+^ salts have been shown to block TRPC channels in vitro, and we therefore tested their potential effect on Ang II‐induced glomerular hyperpermeability. Anesthetized male Sprague‐Dawley rats were infused with Ang II (80 ng kg^–1 ^min^–1^) alone, or together with clemizole or low‐dose La^3+^ (activates TRPC5, blocks TRPC6) or high‐dose La^3+^ (blocks both TRPC5 and TRPC6). Plasma and urine samples were taken during baseline and at 5 min after the start of the infusions and analyzed by high‐performance size‐exclusion chromatography for determination of glomerular sieving coefficients for Ficoll 10–80 Å (1–8 nm). Ang II infusion evoked glomerular hyperpermeability to large Ficolls (50–80 Å), which was ameliorated by clemizole, having no significant effect on glomerular filtration rate (GFR) or Ang II‐mediated increase in mean arterial pressure (ΔMAP). In contrast, high‐ and low‐dose La^3+^ significantly lowered ΔMAP and reduced Ang II‐induced hyperpermeability. Combined, clemizole and low‐dose La^3+^ were less effective at ameliorating Ang II‐induced glomerular hyperpermeability than low‐dose La^3+^ alone. In conclusion, our data show that both clemizole and La3+ are effective against Ang II‐induced glomerular hyperpermeability, with differential effects on blood pressure. Further research using more specific blockers of TRPC5 and TRPC6 should be performed to reveal the underlying mechanisms.

## Graphical abstract

Angiotensin II (Ang II) elicits Ca^2+^ influx into cells, mediated by TRPC5 and TRPC6 (transient receptor potential canonical type 5 and 6). Clemizole and La^3+^ salts have been shown to block TRPC5 and TRPC6 in vitro, and we therefore tested their potential effect on Ang II‐induced glomerular hyperpermeability in anesthetized Sprague‐Dawley rats. Clemizole and La^3+^ effectively reduced Ang II‐induced glomerular hyper permeability, with differential effects on blood pressure.

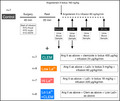

## 1 INTRODUCTION

Inhibitors of the renin‐angiotensin system (RAS), particularly angiotensin (Ang) II receptor blockers (ARBs) and angiotensin‐converting enzyme (ACE) inhibitors, are widely adopted antiproteinuric and antihypertensive agents that prevent the progression of chronic kidney disease (Hsu et al., 2014). However, in conditions where systemic blood pressure is compromised, such as in hypovolemia or shock, RAS inhibitors may critically reduce glomerular filtration pressure by reducing efferent arteriolar tone, leading to AKI (Palevsky PM, Zhang JH, Seliger SL, Emanuele N, Fried LF, and Study VN‐D. Incidence, (2016); Siew & Davenport, 2015).

Podocytes, being highly specialized epithelial cells covering the outer aspect of glomerular capillaries, have a contractile cytoskeleton that appears to play an important role for the integrity of the renal filter (Faul et al., 2007; Schiffer et al., 2015). Dysregulation of Ca^2+^ homeostasis is an early sign of podocyte injury (Tian et al., 2010) and is followed by disruption of the actin cytoskeleton (Faul et al., 2007) indicating a link between Ca^2+^ signaling and cytoskeletal alterations. Importantly, these events in podocytes have been closely associated with proteinuric kidney disease (Schiffer et al., 2015). After binding to its receptor, Ang II elicits Ca^2+^ influx via the classic transient receptor potential channel (TRPC) type 5 and 6 (Tian et al., 2010) triggering a complex cascade of intracellular signaling events (Greka & Mundel, 2011). While the role of TRPC6 in several proteinuric kidney diseases is well‐established (Staruschenko et al., 2019), the role of TRPC5 in glomerular disease is currently being discussed (Pablo & Greka, 2019). Schaldecker *et al* found that genetic knockout or pharmacologic inhibition of TRPC5 protected mice from albuminuria (Schaldecker et al., 2013). Furthermore, Zhou *et al* identified a small‐molecule TRPC5 inhibitor that suppressed proteinuria and podocyte loss in a transgenic rat model of focal segmental glomerulosclerosis (Zhou et al., 2017). More recently, Yu *et al* found beneficial effects of TRPC5 inhibition in experimental mouse models of glomerular disease (Yu et al., 2019). In contrast, Wang and colleagues found that neither TRPC5 overexpression nor activation caused kidney barrier injury during LPS infusion (Wang et al., 2018). Furthermore, Ilatovskaya and co‐authors, using TRPC6‐ and TRPC5/6‐double knockout mice, recently found that TRPC5 did not seem to have an additive effect to TRPC6‐mediated Ca^2+^ influx in podocytes (Ilatovskaya et al., 2018).

We have previously shown that Ang II infusion causes rapid, dynamic changes in the glomerular permeability to macromolecules in Wistar rats, independent of blood pressure (Axelsson et al., 2012, 2013). In the current work, we tested the potential effects of clemizole hydrochloride and La^3+^ salts, inhibitors of TRPC5 (Richter et al., 2014) and/or TRPC6 (Jung et al., 2003), on glomerular hyperpermeability caused by Ang II. We studied sieving coefficients of fluorescein isothiocyanate (FITC) Ficoll, a polydisperse polysaccharide that does not undergo tubular processing and is therefore a robust marker of glomerular permeability.

## 2 METHODS

### 2.1 Animals

Experiments were performed in 37 male 9‐week‐old Sprague‐Dawley rats (Möllegard, Lille Stensved, Denmark) having an average body weight (BW) of 275 g (256 – 302 g) with access to food (Special Diets Services RM1(P) IRR.25 801157) and water *ad libitum*. Approval by the local Animal Ethics Committee at Lund University, Sweden, was obtained for all procedures (Dnr 5.8.18‐05699/2018).

### 2.2 Surgery

Induction of anesthesia was established by means of an intraperitoneal injection of sodium pentobarbital (Pentobarbitalnatrium vet. APL 60 mg/ml), 1.5 mL/kg BW, and maintained, if necessary, by further administration of pentobarbital (~50–100 μL) as needed. The body temperature was maintained at 37°C using a heating pad. A tracheotomy was performed by means of a small incision on the lower anterior aspect of the trachea (intubation using a PE‐240 tube). The tail artery was cannulated (using a PE‐50 cannula) and used for the maintenance of anesthesia and for continuous registration and monitoring of heart rate (HR) and mean arterial blood pressure (MAP) using a data acquisition system (BioPack Systems model MP150 with AcqKnowledge software version 4.2.0, BioPack Systems Inc., Goleta, CA). The left and right internal jugular veins were cannulated (PE‐50) for the iv infusion of Ficoll and the different pharmacological interventions (La^3+^ and clemizole), respectively. For blood sampling, the left carotid artery was cannulated (PE‐50). Access to the left ureter was established via a small abdominal incision (6–8 mm). For urine sampling, the left ureter was cannulated using a small PE‐10 cannula (connected to a PE‐50 cannula). A small dose of furosemide (0.375 mg/kg iv) was given to facilitate the cannulation procedure.

### 2.3 Experimental procedure

**FIGURE 1:**
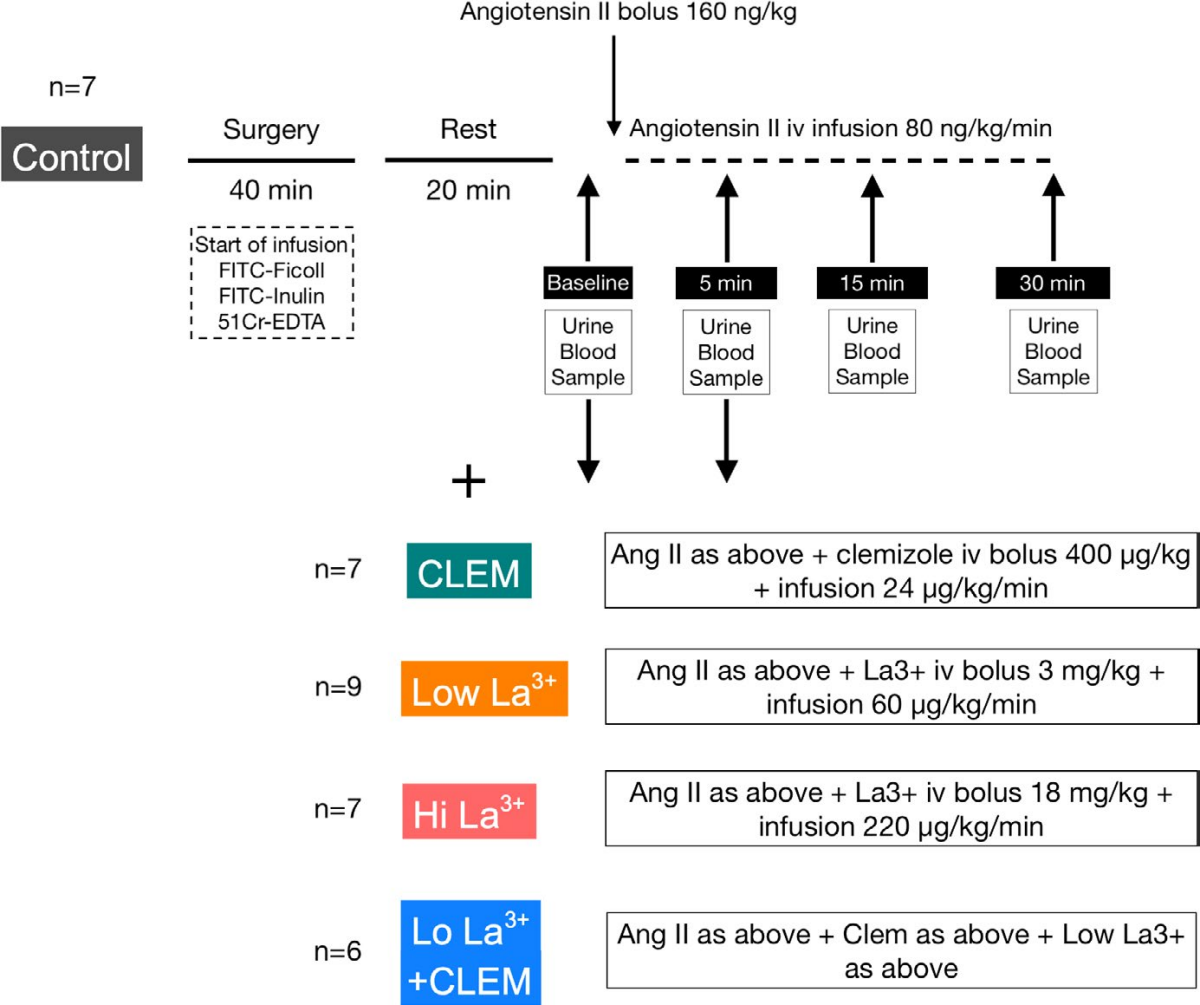
Schematic illustration of the experimental procedures. Control experiments were firstly performed (n = 7) in which angiotensin II was given intravenously after a baseline measurement. Additional measurements of permeability, MAP, and GFR were performed at 5‐, 15‐, and 30‐min post‐administration. Identical experiments were then performed where animals were also given clemizole (n = 7), low‐dose La^3+^ (n = 9), high‐dose La^3+^ (n = 7), and combined clemizole and low‐dose La^3+^ (n = 6) before angiotensin II was administered. In these latter experiments, measurements were only performed at baseline and at 5 min post‐administration of angiotensin II where maximal effects on angiotensin II on MAP and glomerular permeability were observed in the Control experiments

All experimental procedures were initiated by a resting period of 20 min following the cannulation of the left ureter (Figure 1). After an initial 5 min period for control (baseline) measurements (GFR, θ_Ficoll_, MAP, and HR), new measurements were performed following administration of different pharmacological challenges as described in Table 1. Glomerular hyperpermeability was induced by a bolus dose (160 ng/kg) Ang II followed by an iv infusion (80 ng kg^−1 ^min^−1^). The animals were divided into 5 experimental groups: Ang II only (n = 8), Ang II +Hi La^3+^ (n = 7), Ang II +Lo La^3+^ (n = 9), AngII+clemizole (n = 7), and Ang II +Low La^3+^ + clemizole (n = 6). In the AngII+La^3+^ groups, lanthanum(III)chloride heptahydrate (Sigma‐Aldrich, St. Louis, MO) was given as an iv bolus dose (18 mg kg^−1^ for Hi; 3 mg kg^−1^ for Lo) followed by a continuous infusion (220 μg kg^−1 ^min^−1^ iv for Hi; 60 μg kg^−1 ^min^−1^ for Lo). For the AngII+clemizole group, we used clemizole, a potent TRPC5 inhibitor effective against both TRPC5:TRPC5 homomers and TRPC1:TRPC5 heteromers (Richter et al., 2014). Clemizole (Sigma‐Aldrich, St. Louis, MO), was administered as a bolus dose (0.4 mg kg^−1^ iv) and a continuous infusion (24 μg kg^−1 ^min^−1^). The serum concentration of La^3+^ was determined 5 minutes post‐administration of Ang II using inductively coupled mass‐spectrometry (ICP‐MS) using a Perkin Elmer Optima 8300 (Perkin Elmer Instruments, Shelton, CT).

**TABLE 1:** Effects of clemizole and La3+ salts on canonical transient receptor channels (TRPC3/4/5/6)

**Substance**	**TRPC3**	**TRPC4**	**TRPC5**	**TRPC6**
Clemizole	Blocks*	Blocks**	Blocks	Blocks***
Low‐dose La^3+^	Blocks	Activates	Activates	Blocks
High‐dose La^3+^	Blocks	Blocks	Blocks	Blocks

* Clemizole has 8‐fold selectivity of TRPC5 compared to TRPC3 (Richter et al., 2014).

** Clemizole has 6‐fold selectivity for TRPC5 compared to TRPC4 (Richter et al., 2014).

*** Clemizole has 10‐fold selectivity of TRPC5 compared to TRPC6 (Richter et al., 2014).

### 2.4 Determination of the glomerular sieving coefficient for Ficoll (θ_Ficoll_)

During the course of the experiment, a continuous iv infusion (10 ml kg^−1 ^h^−1^) of FITC‐Ficoll (FITC‐Ficoll‐70, 20 µg ml‐1; FITC‐Ficoll‐400, 480 µg ml‐1; FITC‐Inulin, 500 µg ml‐1 and ^51^Cr‐EDTA, 0.3 MBq ml^−1^) was given, initiated by a bolus dose (FITC‐Ficoll‐70, 40 µg; FITC‐Ficoll‐400, 960 µg; FITC‐Inulin 0.5 mg and ^51^Cr‐EDTA 0.3 MBq). This 1:24 mixture of polydisperse Ficoll provides a broad range of molecular sizes. Sieving measurements were performed by means of a 5 min collection of urine and a mid‐point (2.5 min) plasma sample.

### 2.5 Determination of glomerular filtration rate in the left kidney

^51^Cr‐EDTA (Amersham Biosciences, Buckinghamshire, UK) was administered throughout the experimental period (see above). A gamma counter (Wizard 1480, LKP, Wallac, Turku, Finland) was used to detect radioactivity in blood (CPM_blood_) and urine (CPM_urine_) samples. The glomerular filtration rate (for the left kidney) was estimated from the plasma to urine clearance of ^51^Cr‐EDTA.

### 2.6 High‐performance size‐exclusion chromatography (HPSEC)

A HPLC system (Waters, Milford, MA) was applied to quantify the concentration and size distribution of the FITC‐Ficoll samples. Size separation of the plasma and urine samples was accomplished using an Ultrahydrogel 500 column (Waters) connected to a guard column (Waters). A phosphate buffer (0.15 M NaCl, pH 7.4) was used as the mobile phase, driven by a pump (Waters 1525). Fluorescence in the samples was detected at an excitation wavelength 492 nm and emission wavelength of 518 nm. Samples were loaded onto the system using an autosampler (Waters 717 plus). As is described at some length elsewhere (Asgeirsson et al., 2006), the system was calibrated using protein standards and narrow Ficoll standards. The urine FITC‐Ficoll concentration for each size (a_e_) was multiplied by the urine‐to‐plasma FITC‐Inulin concentration ratio to approximate the concentration of Ficoll in primary urine (C_u_). The glomerular sieving coefficient was calculated by dividing C_u_ by the plasma concentration of Ficoll.

### 2.7 Statistical analysis

Values are presented as means ±standard error. Significant differences were assessed using a repeated measures ANOVA on aligned rank transformed data (using ARTool version 0.10.5), essentially performing a non‐parametric test using parametric methods (Wobbrock et al., 2011). Pairwise comparisons were made with Wilcoxon signed rank tests. We used an alpha level (probability of type I error) of 0.05 and a beta level (probability of type II error) of 0.20 unless otherwise specified. Bonferroni corrections for multiple comparisons were made when applicable. A previous power analysis showed that four (n = 4) was the minimal number of animals needed to detect a difference in sieving coefficients by a factor of at least 2 (Dolinina et al., 2018). Statistical analysis was performed using R version 3.5.1 for macOS (The R Foundation for Statistical Computing).

## 3 RESULTS

### 3.1 Clemizole ameliorates ang II‐induced glomerular hyperpermeability in vivo without significant hemodynamic effects

**FIGURE 2:**
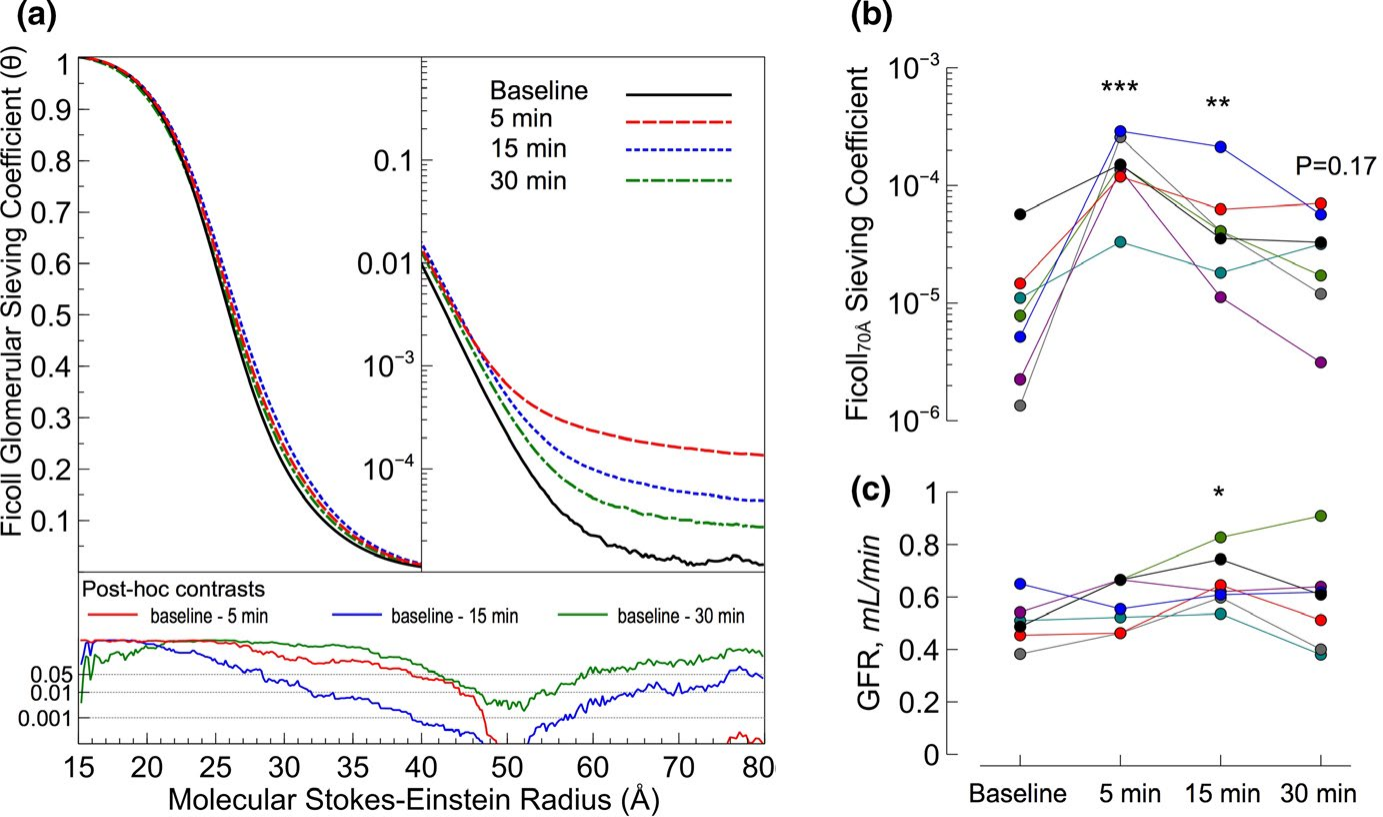
(a) Glomerular sieving coefficients vs. molecular Stokes–Einstein radius for Ficolls ranging from 15 Å (about the size of FITC‐inulin) to 80 Å at baseline and at the different timepoints post‐administration of AngII. In a graph below, the plotted sieving curve are P‐values from post hoc tests comparing sieving coefficients post‐administration vs. baseline. (b) Glomerular sieving coefficients for Ficoll_70 Å_ and (c) glomerular filtration rate at baseline and 5, 15, and 30 min after AngII administration

**FIGURE 3:**
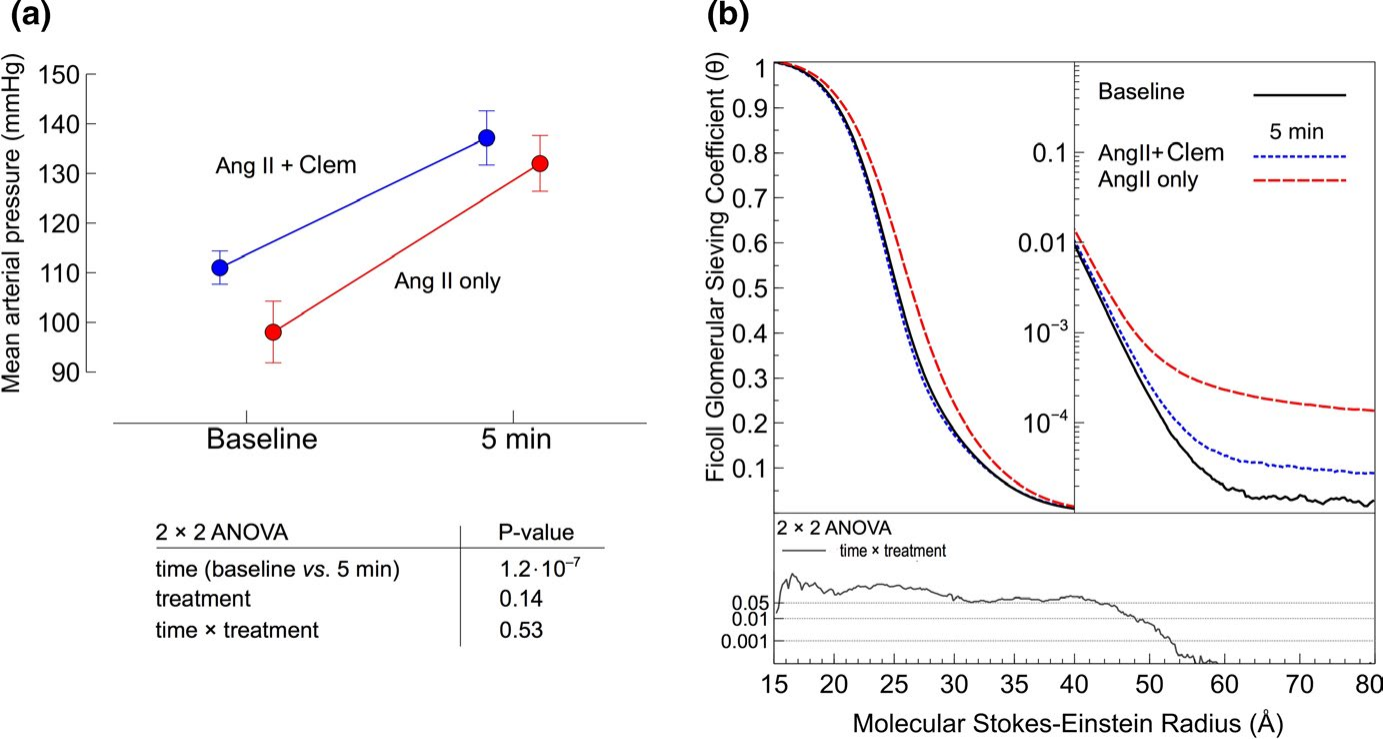
(a) Mean arterial pressure at baseline and 5 min post‐Ang II administration (red) for the Ang II only group (n = 7) and Ang II +Clemizole (Clem) group (blue) (n = 7). A 2 × 2 ANOVA revealed no interaction between treatment (Ang II only *vs*. Ang II +Clemizole) and time (baseline *vs*. 5 min) showing that clemizole did not affect the hemodynamic effects of angiotensin II. (b) Sieving coefficients at baseline and 5 min for the Ang II +Clemizole group (black and blue lines) compared with the Ang II only group. A significant interaction (treatment ×time) indicates that TRPC5 inhibition significantly ameliorates Ang II‐mediated glomerular hyperpermeability for Ficolls >45 Å. * *p* < 0.05 (Wilcoxon), ** *p* < 0.01 (Wilcoxon), *** *p* < 0.001 (Wilcoxon)

Ang II elicited rapid, dynamic elevations in glomerular sieving coefficients (θ) for large Ficolls >45 Å, being increased more than an order of magnitude after 5 min (Figure 2a). Small, transient, increments in θ also occurred for Ficolls <45 Å at 15 min. In addition, at the same time point, the left kidney glomerular filtration rate was significantly higher compared to baseline (Figure 2c). In contrast to the changes in permeability, mean arterial pressure remained elevated through the entire experimental period (Table 2), similar to previous results (Axelsson et al., 2012). Previous work has also demonstrated that the effects of Ang II on glomerular permeability and mean arterial pressure can be completely abrogated by angiotensin receptor blockade (Axelsson et al., 2012). In contrast, we find that clemizole did not significantly affect the hemodynamic pressor effects of Ang II with the increase in MAP being very similar between Ang II only and Ang II +clemizole (Figure 3a). Clemizole did however effectively reduce the changes in glomerular permeability caused by Ang II (Figure 3b) for Ficolls >45 Å, similar to Ang II blocking (Axelsson et al., 2012).

**TABLE 2:** Mean arterial pressure and heart rate after Ang II administration only.

Parameter	Baseline	5 min	15 min	30 min
MAP, mmHg	98 ± 6	132 ± 6***	134 ± 5***	134 ± 4***
Heart rate, min^−1^	275 ± 12	263 ± 15	264 ± 17	268 ± 20

*** *p* < 0.001 compared to baseline

### 3.2 Effects of La^3+^ on hemodynamic effects and glomerular hyperpermeability induced by angiotensin II

**FIGURE 4:**
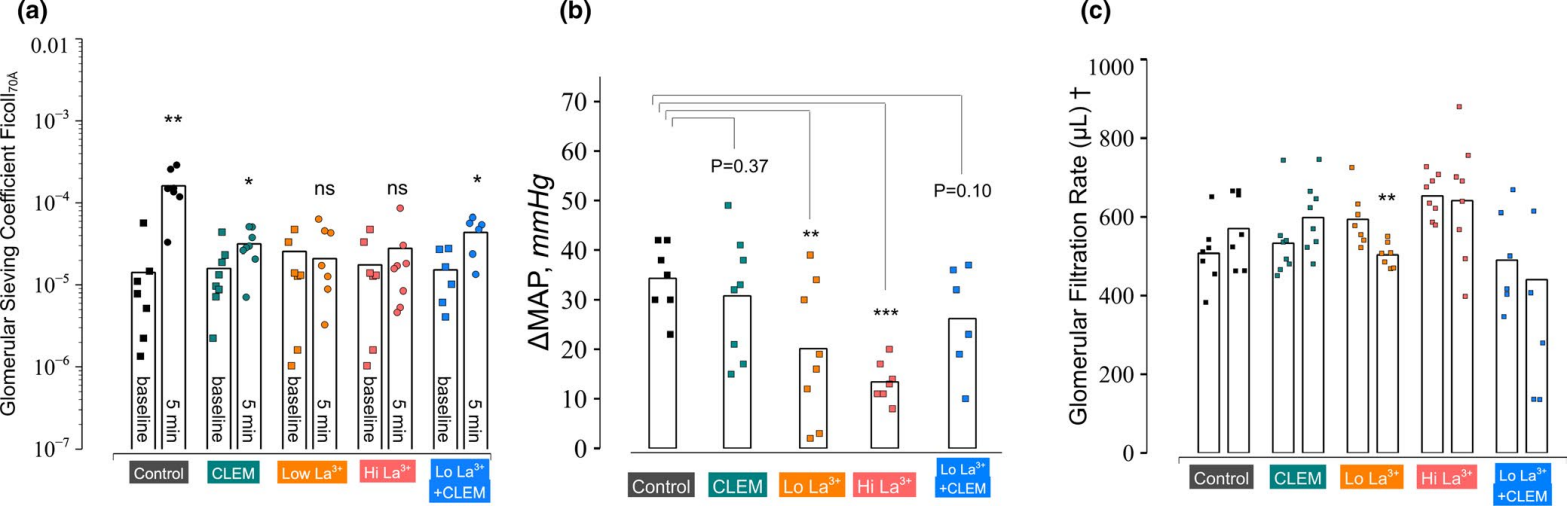
(a) Glomerular sieving coefficients for Ficoll_70 Å_ at baseline and 5 min after Ang II administration for the different experimental groups. (b) Difference in mean arterial pressure between baseline and 5 min post‐Ang II. Statistical differences are between Ang II only and the groups combining Ang II with other substances. (c) Glomerular filtration rate (GFR) for the left kidney in the different groups. For high‐dose La^3+^, there was a significant decrease in GFR between baseline and 5 min

Lanthanides like La^3+^ and Gd^3+^ have been shown to activate TRPC5 at micromolar concentrations while at the same time inhibiting TRPC6 in a dose‐dependent manner (Jung et al., 2003). Here we firstly administered iv La^3+^ in a low dose (60 μg kg^−1 ^min^−1^ giving a serum concentration of ~0.3 mM), thus activating the TRPC5 channel rather than blocking it while blocking the TRPC6 channel followed by separate experiments using millimolar concentrations of La^3+^ (220 μg kg^−1 ^min^−1^ giving a serum concentration of ~1.6 mM) blocking both the TRPC5 and TRPC6 channel. Both La^3+^ concentrations were effective in blocking the glomerular hyperpermeability response induced by Ang II (Figure 4a). Both low‐ and high‐dose La^3+^ effectively counteracted the increments in mean arterial pressure induced by Ang II (Figure 4b). Lastly, we administered both low‐dose La^3+^ and clemizole in a separate group. The combined therapy appeared less effective at reducing MAP than low‐dose La^3+^ alone. Accordingly, in a separate analysis, we tested the interaction treatment × time on the sieving coefficient of Ficoll_70 Å_ between the low‐dose La^3+^ and the combined La^3+^ + clemizole group. We rejected the null hypothesis at the 1% level (*F*_1,13_ = 9.4, *p* = 0.009) showing that the combined treatment was inferior in its ability reduce the leakage of large Ficoll_70 Å_ across the glomerular filter compared to low‐dose La^3+^ only suggesting that concomitant administration of clemizole potentiates the hyperpermeability reducing effect of low‐dose La^3+^.

## 4 DISCUSSION

Our data demonstrate that clemizole effectively ameliorates Ang II‐induced glomerular hyperpermeability without significantly affecting systemic blood pressure. These findings are in line with results both by Zhou et al (Zhou et al., 2017) (using a more specific TRPC5 blocker) and those by Ilatovskaya et al (Ilatovskaya et al., 2017). However, we cannot from the current experiments conclude that the observed effects are due to TRPC5‐inhibition since clemizole is also a weak TRPC6 blocker and a first‐generation H_1_ receptor blocker. Additionally, experiments performed in whole animals may include other unknown or unmeasured systemic phenomena that may influence results since TRPC5 and TRPC6 are present in many other organs. In contrast, the strength of whole animal physiology is the ability to study actual physiological phenomena that are close to those in humans. Thus, the present findings may have translational value inasmuch as clemizole hydrochloride appears to act as a “glomerular angiotensin II receptor blocker” in Sprague‐Dawley rats which may have important implications for further clinical and experimental studies. Clemizole hydrochloride has for long been used as a peroral anti‐histamine and thus may be more easily tested as a potential antiproteinuric agent in clinical studies compared to drugs with an unknown safety profile. Another limitation in the current study is that the anesthesia used (pentobarbital) may have influenced hemodynamic parameters. In particular, the choice of anesthesia may affect how the animals react to vasoactive substances such as AngII (and La^3+^). Therefore, future studies using other anesthetics should be performed to see if the present results on MAP and GFR can be replicated.

We also found that La^3+^ salts reduced Ang II‐induced glomerular hyperpermeability in a dose‐dependent manner. In addition, La^3+^ counteracted the vasopressor effects of Ang II, which may be an effect of TRPC6‐inhibition. Indeed, several lines of evidence have indicated that TRPC6‐mediated Ca^2+^ influx in vascular smooth muscle cells is associated with vasoconstriction (Ghosh et al., 2017). In podocytes, TRPC6 signaling is associated with increased contractility and stress fiber formation, while TRPC5 appears to reduce contractility and stress fiber formation, promoting cell motility through the formation of lamellipodia (Tian et al., 2010). However, while Ang II‐induced Ca^2+^ influx seems to occur mainly via TRPC5 and TRPC6 in podocytes (Tian et al., 2010), the observed effects on mean arterial pressure may be due to the effects of La^3+^ on other channels and future experiments should be performed to elucidate the mechanisms behind the antihypertensive effects of La^3+^ salts, for example by using a more specific TRPC6 inhibitor. Interestingly, combining clemizole and low‐dose La^3+^ had significantly less effect on glomerular hyperpermeability compared to low‐dose La^3+^ alone. While this finding may appear counterintuitive, it could possibly be the result of an antagonistic relationship between these channels in podocytes (Tian et al., 2010). We have previously demonstrated that blocking either the TRPC5‐associated GTP‐ase Rac‐1 or the TRPC6‐associated GTP‐ase RhoA is also effective to counteract Ang II effects on glomerular permeability. Likely, blocking either RhoA‐kinase or TRPC6 has the same effect as blocking either Rac‐1‐kinase or TRPC5. One possible interpretation of our current results is that blocking just one channel is more effective than blocking both channels, supporting the view that it is a concerted and mutually inhibitory action of both Rac‐1 and RhoA signaling pathways that governs the glomerular hyperpermeability response (See (Greka & Mundel, 2011)), rather than the sole action of one of the pathways. Whatever the underlying mechanism, the current results are difficult to reconcile with a dose‐response effect on the basis of TRPC6‐blocking only, since then the combination of clemizole and low‐dose La^3+^ should be at least as effective as either substance alone.

Glomerular hyperpermeability can be induced by a number of pharmacological and experimental challenges (Axelsson et al., 2012; Dolinina et al., ,^2016^, ^2018^, ^2019^) often being of a transient nature such that the permeability approaches baseline levels after the injurious stimuli are removed. While proteinuria is often associated with structural alterations in the renal filter, such reversible alterations imply that the structural changes occurring in the GFB are of a brief and non‐persistent nature. While there is good evidence that podocytes play a key role in proteinuric disease, the mechanisms by which they keep the renal filter tight are still obscure. Very recently, it was hypothesized that podocytes, via their contractile cytoskeleton, maintain a tension compressing the GBM in the radial direction keeping the meshwork of the GBM in an organized state (Fissell & Miner, 2018). According to this hypothesis, loss of tension will disorganize the GBM, reducing its size‐selective properties, leading to leakage of macromolecules across the filter. In terms of a pore model of glomerular permeability (Oberg & Rippe, 2014), this would be equivalent to increasing the average pore radius or widening the pore size distribution.

We have previously identified several potential pharmacological targets downstream of the Ang II receptor (Axelsson et al., 2012, 2013). However, since TRPC channels are partly extra‐cellular, they are more easily targeted by pharmacological interventions. A limitation in the current study is the non‐selective nature of La^3+^ salts and also clemizole, meaning that the observed effects may be due to blocking of other TRP channels. Further research could be performed with more selective blockers of TRP channels to elucidate the underlying mechanisms.

## CONFLICT OF INTERESTS

The authors declare no competing interests pertaining to the current article.

## AUTHOR CONTRIBUTIONS

JD, AR and CMÖ conceived and designed the experiments. JD and AR performed experiments. JD and CMÖ analyzed the data, and drafted the manuscript. All authors discussed the results, and approved the final version of the manuscript.
